# Characterization of a New *CDC73* Missense Mutation that Impairs Parafibromin Expression and Nucleolar Localization

**DOI:** 10.1371/journal.pone.0097994

**Published:** 2014-05-19

**Authors:** Giulia Masi, Maurizio Iacobone, Alessandro Sinigaglia, Barbara Mantelli, Gianmaria Pennelli, Ignazio Castagliuolo, Giorgio Palù, Luisa Barzon

**Affiliations:** 1 Department of Molecular Medicine, University of Padova, Padova, Italy; 2 Istituto Oncologico Veneto IRCCS (Istituto di Ricovero e Cura a Carattere Scientifico), Padova, Italy; 3 Department of Surgery, Oncology and Gastroenterology, University of Padova, Padova, Italy; 4 Department of Medicine, University of Padova, Padova, Italy; CNR, Italy

## Abstract

Mutations of the Cell Division Cycle 73 (*CDC73)* tumor suppressor gene (previously known as *HRPT2),* encoding for parafibromin, are associated with the Hyperparathyroidism-Jaw Tumor (HPT-JT) syndrome, an autosomal dominant disease whose clinical manifestations are mainly parathyroid tumors and, less frequently, ossifying fibromas of the jaws, uterine and renal tumors. Most mutations of *CDC73* are nonsense or frameshift, while missense mutations are rare and generally affect the N-terminal domain of parafibromin, a region that is still poorly characterized. The aim of this study was to characterize a novel somatic *CDC73* missense mutation (Ile60Asn) identified in the mandibular tumor of a HPT-JT patient carrying a germline *CDC73* inactivating mutation. Immunostaining of the tumor showed reduced nuclear parafibromin immunoreactivity. Western blotting and confocal microscopy of transfected cells demonstrated that the Ile60Asn mutant parafibromin was less expressed than the wild-type protein and exhibited impaired nucleolar localization. Treatment of transfected cells with translation and proteasome inhibitors demonstrated a decreased stability of the Ile60An mutant, partially due to an increase in proteasomal degradation. Overexpression of the Ile60Asn mutant led to increased cell proliferation and to accumulation in the G2/M phase of cell cycle. Moreover, mutant parafibromin lost the ability to down-regulate c-myc expression. In conclusion, our study shows that a missense mutation in the N-terminus of parafibromin, identified in an ossifying fibroma from a HPT-JT patient, stimulated cell proliferation and impaired parafibromin expression and nucleolar localization, suggesting a relevant role of the N-terminal domain for parafibromin function.

## Introduction

The Hyperparathyroidism-Jaw Tumor (HPT-JT) syndrome is an autosomal dominant disease whose first clinical manifestation is usually the occurrence of parathyroid tumors, that are malignant in 15% of cases; in addition, 25–50% of patients may develop ossifying fibromas of the jaws and benign or malignant renal and uterine tumors [1]. The ossifying fibromas of mandible and maxilla occurring in HPT-JT patients are histologically distinct from the osteoclastic ‘brown tumors’ typical of primary hyperparathyroidism, and may occur at very early age [1].

Mutations of the Cell Division Cycle 73 (*CDC73*) gene, previously known as *HRPT2*, have been identified in >80% of the reported HPT-JT families [2]. *CDC73* encodes for parafibromin, a 531 amino acids protein that acts in the context of the RNA polymerase II associated factor 1 (PAF1) transcriptional regulatory complex [3]. The human PAF1 complex associates with RNA polymerase II throughout the entire coding region of transcriptionally active genes, indicating roles in initiation, elongation and post-transcriptional events [2]. Parafibromin has many different functions: among the most relevant for its tumor suppressor activity, there are repression of cyclin D1 [4]–[5] and inhibition of the c-myc proto-oncogene [6]. Parafibromin has also been reported to activate the Wnt signaling through interaction with β-catenin [7] in its dephosphorylated form [8].

Despite most of the identified *CDC73* mutations reported to date are frameshift or nonsense [1], large or whole-gene deletions have also been described [9]–[11]. Missense mutations are rare and generally affect residues located in the still poorly characterized N-terminus of parafibromin. Crystallographic determination of the structure of the C-terminal domain of the yeast homologue of parafibromin, Cdc73, revealed a fold highly similar to that of the small GTPase Ras [12]–[13]. Unfortunately, no structural data on the N-terminal domain of the protein are available to date. Moreover, parafibromin interaction domains with known proteins have been identified in the C-terminal domain in amino acids between 200 and 413, but not in the N-terminal domain [1].

In the present study we characterized a somatic missense mutation (*i.e.*, Ile60Asn) located in the N-terminal domain of *CDC73,* identified in a mandibular ossifying fibroma that occurred in a HPT-JT patient. This mutation led to decreased parafibromin expression, impaired its nucleolar localization, and determined an increase in cell proliferation, thus suggesting a relevant role of the N-terminal domain for parafibromin function.

## Patient and Methods

### Ethics Statement

Written informed consent for the collection of personal, genetic, and clinical data was obtained from the patient. This study was approved by the Ethics Committee of Padova University Hospital.

### Patient

The patient investigated in this study was previously described as belonging to a kindred with familial isolated hyperparathyroidism carrying a germline *CDC73* c.(136_144)del5 germline mutation [14]–[15].

Patient’s past medical history was characterized by the occurrence of two parathyroid adenomas and of multiple endometrial hyperplastic polyps. At the age of 28 years, the patient received a diagnosis of HPT-JT because of the occurrence of a 3-cm ossifying fibroma in the left mandible, confirmed by histopathological examination of the resected tumor.

### *CDC73* Sequencing and Mutation Analysis

DNA purification from tissue specimen and mutation analysis of the *CDC73* gene were performed as previously reported [16]. The MutationTaster tool (www.mutationtaster.org) was consulted to predict *in silico* the pathogenicity of the Ile60Asn mutation. Briefly, MutationTaster [16] is a free application for the evaluation of the disease-causing potential of a DNA sequence alteration. This prediction tool analyzes evolutionary conservation, splice-site changes, loss of protein features and changes that affect mRNA amount. Test results are evaluated by a Bayes classifier, which predicts the disease potential, choosing among three prediction models (*i.e.* synonimous alteration, single amino acid alteration, alteration causing a complex change in the amino acid sequence).

### Anti-parafibromin Immunohistochemistry

Immunohistochemical staining was performed on formalin-fixed, paraffin-embedded tissue sections using an automated system (Bond-maX, Leica, Newcastle Upon Tyne, UK). Sections were incubated for 1 h at room temperature with a mouse monoclonal antibody (SC-33638, diluted 1∶200, Santa Cruz Biotechnology Inc., Santa Cruz, CA) raised against a peptide corresponding to amino acids 87–100 of parafibromin. The staining was visualized with 3, 3′-diaminobenzidine and the slides were counterstained with haematoxylin. Normal human parathyroid tissue and a sporadic parathyroid adenoma with wild-type *CDC73* were used as positive controls.

### Cell Lines, Plasmids and Transfections

Transient transfection experiments were performed by using Lipofectamine 2000 Reagent (Life Technologies, Carlsbad, CA) in HEK293A cells (Qbiogene, Carlsbad, CA) and HeLa cells (ATCC, Manassas, VA) with plasmid CDC73-GFP, mediating the expression of parafibromin with a C-terminal fusion of *turbo*-Green Fluorescent Protein (tGFP; OriGene Technologies, Rockville, MD); the empty pCMV6-AC-GFP vector (OriGene Technologies) was employed as a negative control. The Ile60Asn *CDC73* point mutant derivative was generated by site-directed mutagenesis of CDC73-GFP using the QuikChange II Site-Directed Mutagenesis Kit (Agilent Technologies, Santa Clara, CA). The transfection efficiency with the empty pCMV6-AC-GFP vector, as estimated by fluorescence microscopy, was 70–80% in all the experiments.

### Real-time RT-PCR

For real-time RT-PCR, RNA was purified with a RNeasy kit (Qiagen GmBH, Germany), treated with DNase and retro-transcribed with random hexamers and MuLV reverse transcriptase (Life Technologies). *CDC73* mRNA level was measured with a TaqMan Gene Expression assay (Hs00363810_m1, Life Technologies). Relative quantification was calculated through the 2^−ΔΔct^ method normalized to *RNase P* mRNA. Oligonucleotide primers and probes for *RNase P* real-time RT-PCR were as follows: forward, 5′-agatttggacctgcgagcg-3′; reverse, 5′-gagcggctgtctccacaagt-3′; probe, 5′-FAM-ttctgacctgaaggctctgcgcg-TAMRA-3′.

### Western Blotting

For Western blot analysis, proteins were purified from cells by using *M-PER Extraction Reagent* (Thermo Scientific, Waltham, MA) supplemented with a protease and phosphatase inhibitor cocktail (Thermo Scientific). Total proteins were quantified with a *BCA Protein Assay Kit* (Thermo Scientific). The primary antibodies used in this study were: anti-*CDC73* (A264, Cell Signaling Technology, Danvers, MA) at 1∶1000 dilution, anti-tGFP (TA150041, OriGene Technologies) at 1∶10000 dilution, anti-cyclin D1 (SP4, Abcam, Cambridge, UK) at 1∶500 dilution, anti-c-myc (N-262, Santa Cruz Biotechnology Inc.) at 1∶500 dilution, and anti-β-actin (AC-15, Sigma-Aldrich, Saint Louis, MO) at 1∶10000 dilution.

### Analysis of Proteasomal Degradation and of Steady-state Level of Mutant Parafibromin

Twenty-four hours after transfection with tGFP-expressing plasmid and wild-type or mutant *CDC73* plasmid vectors, HeLa cells were treated with the MG132 (25 µM) inhibitor of proteasome activity (Sigma-Aldrich) or with Opti-MEM medium (Life Technologies) as a negative control. Six hours after MG132 treatment, cells were harvested and levels of wild-type and mutant parafibromin were assessed by Western blotting.

The protein translation inhibitor cycloheximide (CHX, Sigma-Aldrich) was added at 20 µM concentration 24 h after transfection. HeLa cells were harvested at 1, 2, 4 and 6 h after treatment and total cell proteins were extracted. The steady-state level of transfected parafibromin was determined by western blotting as described above.

### Immunofluorescence and Laser Confocal Imaging

For co-localization studies, cells transfected with either the empty tGFP-expressing plasmid or wild-type or mutant *CDC73* plasmid vectors were stained with an anti-nucleophosmin antibody (anti-B23 (C-19)-R antibody, Santa Cruz Biotechnology, Inc.). Nuclei were stained with Toto-3 Iodide dye (Life Technologies). Images were acquired on a Leica TCS-SP2 confocal microscope (Leica Microsystems GmbH, Mannheim, Germany). The three dyes were acquired separately to minimize crosstalk. Images were processed using the Leica TCS-SP2 software.

### Cell Cycle and Apoptosis Analysis by Flow Cytometry

To investigate the effect of the *CDC73* Ile60Asn mutation on cell cycle distribution, HEK293A cells were analyzed at 48 h after transfection by flow cytometry for propidium iodide staining. Cells were also stained with the viability dye Calcein Violet 450 AM (eBioscience, San Diego, CA).

For apoptosis analysis, propidium iodide and APC-conjugated annexin V staining (eBioscience) were evaluated at 48 h after transfection. Samples were tested in a BD LSR II Cytofluorimeter (BD Biosciences, San Josè, CA), and data were analyzed with the ModFit LT (BD Biosciences) and the FlowJo (Treestar Inc., Ashland, OR) software. Results from flow cytometry were statistically analysed by the Mann-Whitney test using SPSS v19 software. A *P*<0.05 was considered to be statistically significant.

### Analysis of Cell Proliferation

HeLa cells were seeded in 6-well plates at a density of 3×10^5^ cells/well and transfected as described above. Twenty-four, 48 and 72 h after transfection, cells were harvested and live cells were counted on a counting chamber (Kova Glasstic Slide 10, Hycor Biomedical Inc., Garden Grove, CA) following manufacturer’s instructions.

## Results

### A Novel Somatic Missense Mutation in the *CDC73* Gene

**Figure 1:**
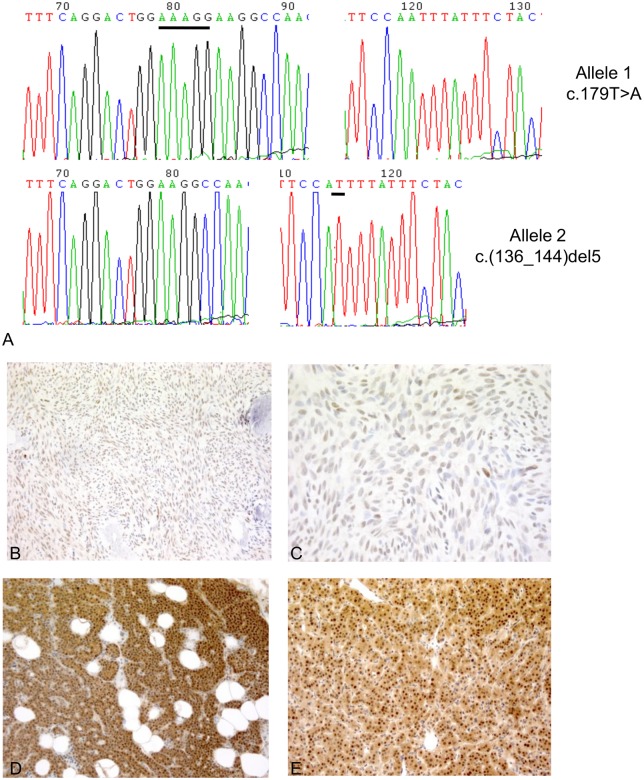
*CDC73* sequencing and anti-parafibromin immunohistochemistry in the mandibular tumor. **A.** Electropherograms of *CDC73* exon 2 PCR amplicons from tumor DNA after subcloning. Upper panel: allele carrying only the somatic c.179 T>A transversion; lower panel: allele carrying the germline c.(136_144)del5 frameshift mutation. The nucleotides in the wild- type sequences involved in the frameshift deletion and in the single nucleotide substitution are underlined with black lines. **B**, **C**. Anti-parafibromin immunostaining in the ossifying fibroma of the jaw. Original magnifications: 10X (A) and 63X (B). **D**, **E**. Anti-parafibromin immunostaining in positive controls: human normal parathyroid tissue (**D**) and sporadic parathyroid adenoma with wild-type *CDC73* (**E**). Original magnification: 10X.

Sequencing of the whole *CDC73* gene in the jaw tumor demonstrated the presence of a heterozygous T>A transversion at position 179 (c.179 T>A) in exon 2, leading to an Ile60Asn substitution. This mutation was demonstrated to be somatic due to its absence in peripheral blood DNA. The presence of the known germline frameshift c.(136_144)del5 mutation in exon 2 was confirmed in the tumor specimen. Subcloning of *CDC73* exon 2 amplicon from tumor DNA into a plasmid vector and sequencing of different clones demonstrated that the c.(136_144)del5 and c.179 T>A mutations were on different alleles (Figure 1A). The Ile60Asn change was predicted to be “disease causing” with a probability of the prediction >0.99 by the MutationTaster software [16]. A conservation analysis through alignment of human parafibromin with the amino acid sequence homologues of ten other species demonstrated that the isoleucine at position 60 was highly conserved, with *F. rubripes* as the evolutionary lowest species where the amino acid is present. Moreover, in *C.elegans* and *D.melanogaster*, the residue at position 60 is a leucine, thus retaining the hydrophobic properties of isoleucine.

### The Ile60Asn Parafibromin Mutant is Less Expressed than Wild-type Protein

**Figure 2:**
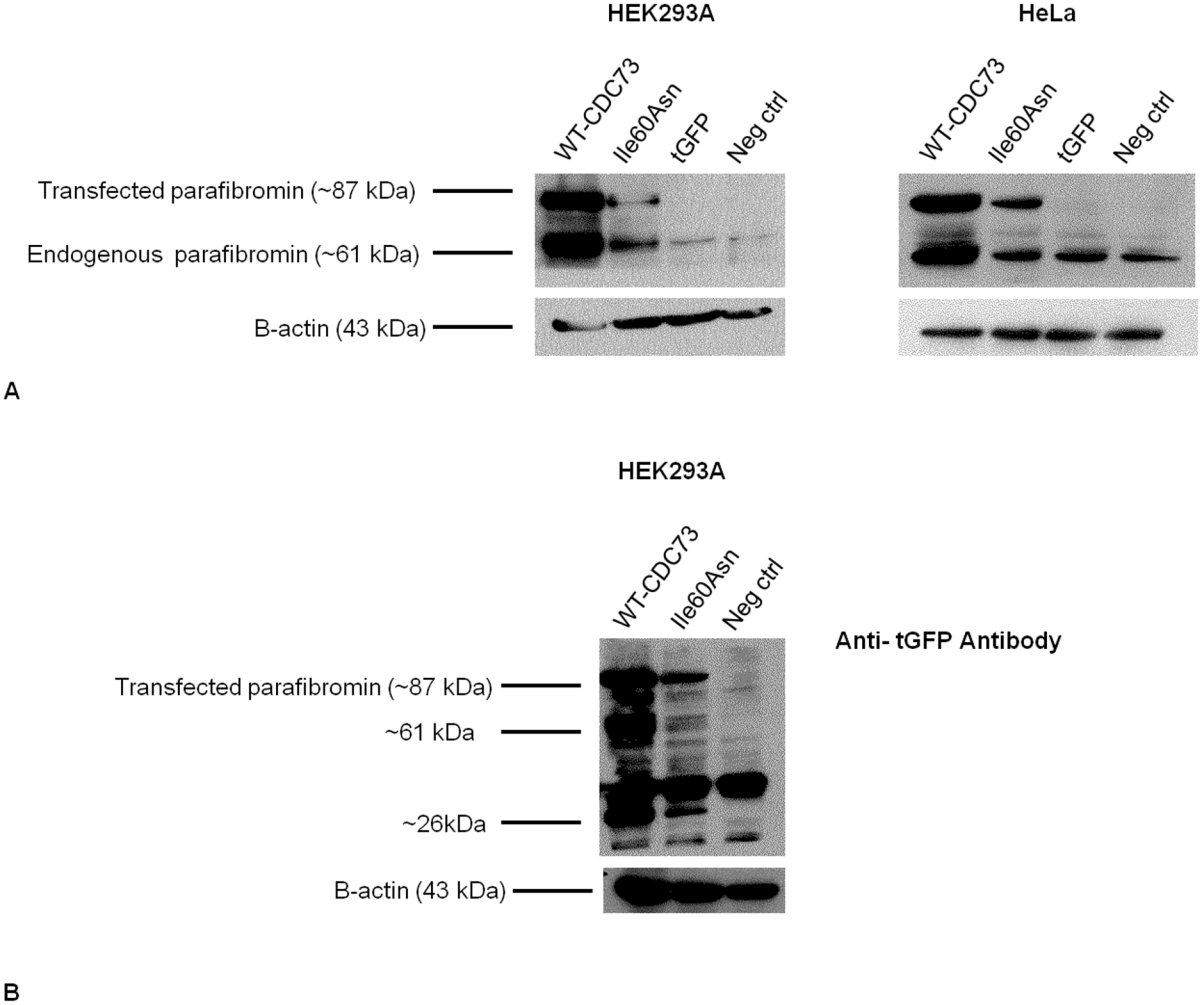
Western blotting for parafibromin and tGFP 48 hours post-transfection. Immunoblotting for parafibromin in HEK293A (**A**) and HeLa cells (**B**) transfected with Ile60Asn-tGFP-fused parafibromin **(Ile60Asn**), wild-type-tGFP-fused parafibromin (**WT-CDC73**) or empty vector (**tGFP**). Nontransfected cells (**Neg ctrl**) have been used as a negative control. **C**. Western blotting with an anti-tGFP antibody has been performed as a control on Ile60Asn and wild-type-parafibromin-transfected cells and on negative control cells. ∼61 kDa: band showing proteolytic degradation of the exogenous parafibromin-tGFP, which generated a protein fragment of approximately the same size of endogenous parafibromin. ∼26 kDa: band corresponding to unconjugated tGFP molecular weight.

**Figure 3:**
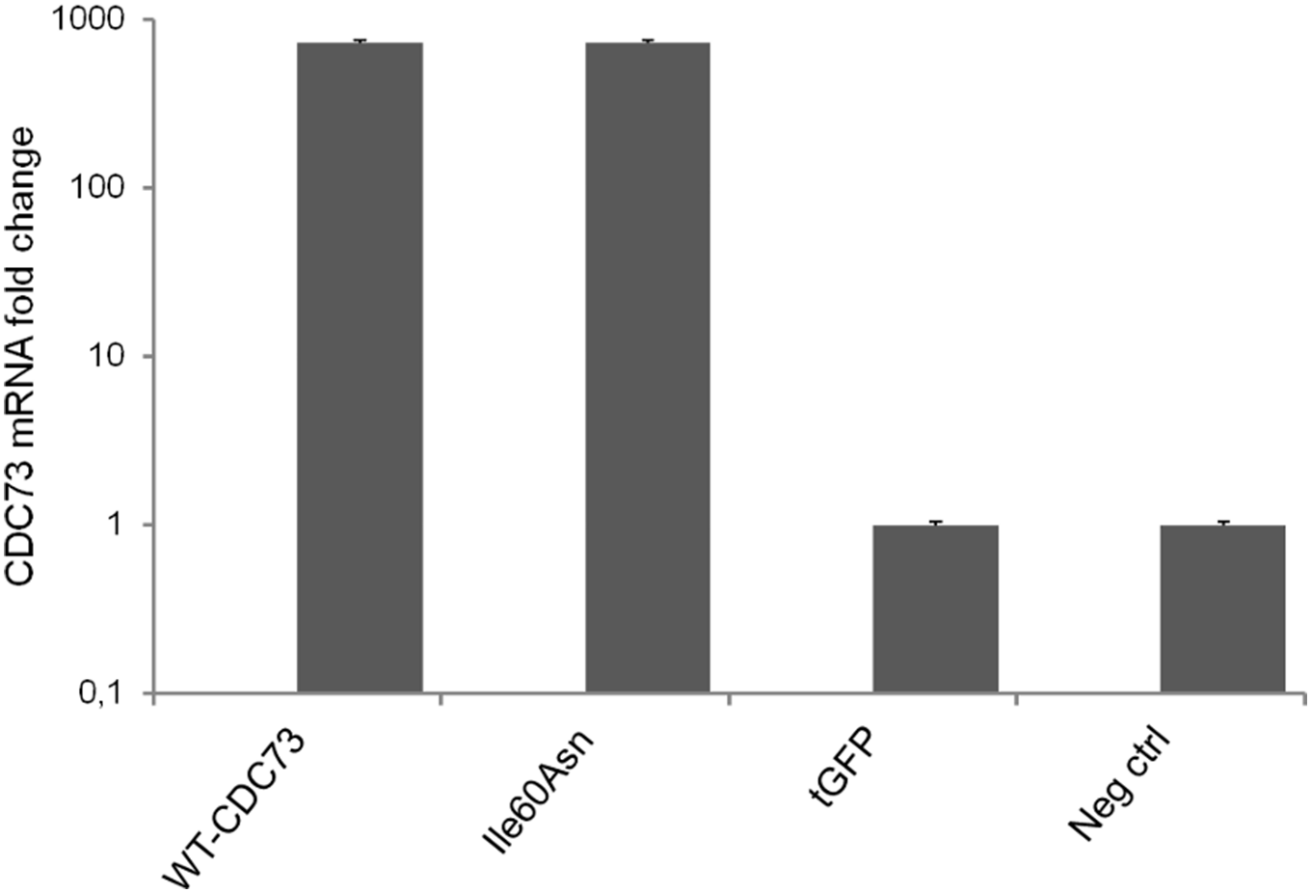
Real-time RT-PCR for *CDC73* mRNA in transfected and nontransfected HEK293A cells. Histogram representing the *CDC73* mRNA fold change in HEK293A cells transfected with Ile60Asn-tGFP-fused parafibromin (**Ile60Asn**), wild-type-tGFP-fused parafibromin (**WT-CDC73**) or empty vector (**tGFP**). Nontransfected cells (**Neg ctrl**) have been used as a negative control.

Immunohistochemical investigation of the mandibular tumor showed a faint but diffuse nuclear parafibromin immunoreactivity (Figures 1B and 1C), indicating that the Ile60Asn mutation did not completely abolish parafibromin expression. Positive controls (normal parathyroid tissue and sporadic parathyroid adenoma without *CDC73* mutations) exhibited an intense nuclear and cytoplasmic positivity (Figures 1D and 1E). Western blot analysis of lysates of HEK293A and HeLa cells transfected to transiently express either wild-type or Ile60Asn mutant parafibromin as fused to tGFP, demonstrated that the expression levels of mutant parafibromin were markedly lower than those of wild-type parafibromin (Figure 2A). The apparent increase in endogenous parafibromin in HEK293A cells transfected with wild-type and mutant *CDC73*-tGFP shown in Figure 2B could be explained by a partial proteolytic degradation of the exogenous parafibromin-tGFP, which generated a protein fragment of approximately the same size (61 kDa) of endogenous parafibromin (Figure 2B). As demonstrated by real-time RT-PCR, cells transfected with either wild-type or Ile60Asn parafibromin expressed *CDC73* transcript at equal level, with a 700-fold increase with respect to nontransfected cells (Figure 3), thus implying a post transcriptional down-regulation of the mutant gene.

**Figure 4:**
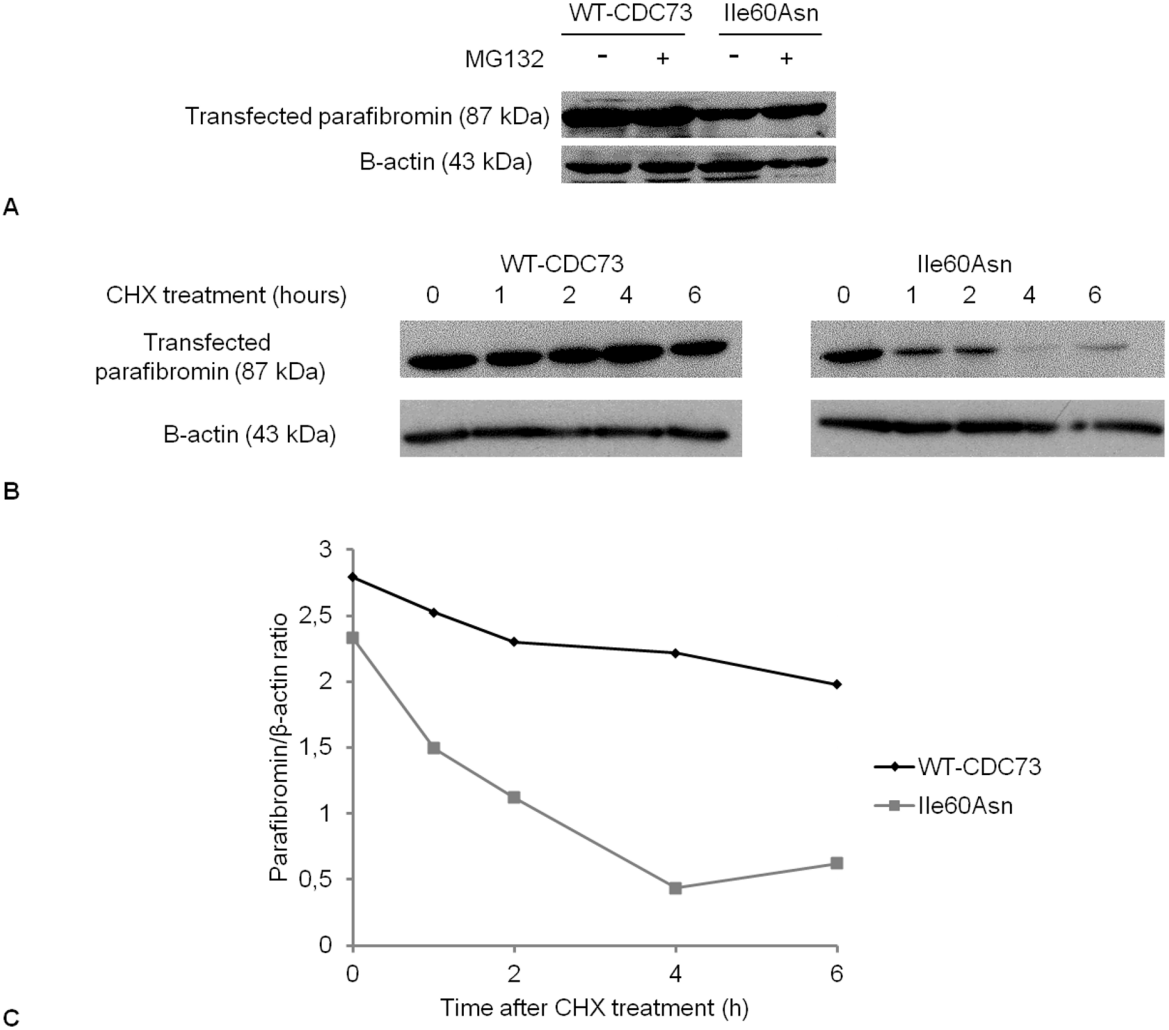
Western blotting analysis of proteasomal degradation and stability of Ile60Asn parafibromin. **A**. Western blotting for parafibromin (with anti-*CDC73* antibody) in HeLa cells in absence (−) or in presence (+) of the proteasome inhibitor MG132. **B**. Western blotting for parafibromin in HeLa cells after treatment with CHX (0 = no treatment) and harvesting at the indicated time points. **C**. Graphical representation of the stability of wild-type parafibromin (WT-CDC73) and Ile60Asn mutant at different time points after treatment with CHX.

To evaluate if the reduction of Ile60Asn parafibromin expression was due to increased proteasomal degradation or if protein stability was decreased, expression of wild-type and mutant parafibromin was analyzed in transfected HeLa cells after treatment with the proteasome inhibitor MG132 or with the translation inhibitor CHX, respectively. Densitometric analysis of western blot experiments showed that treatment with the proteasome inhibitor increased mutant parafibromin level of approximately 40%, while wild-type parafibromin showed no sensitivity to MG132 treatment (Figure 4A).

Exposure to the translation inhibitor for 6 h slightly dropped wild-type parafibromin level, while markedly decreased the Ile60Asn mutant expression. In particular, after 4 h of exposure to CHX, the level of mutant parafibromin was reduced by 73% (Figures 4B and 4C).

### The Ile60Asn Mutation Impairs Nucleolar Localization of Parafibromin

**Figure 5:**
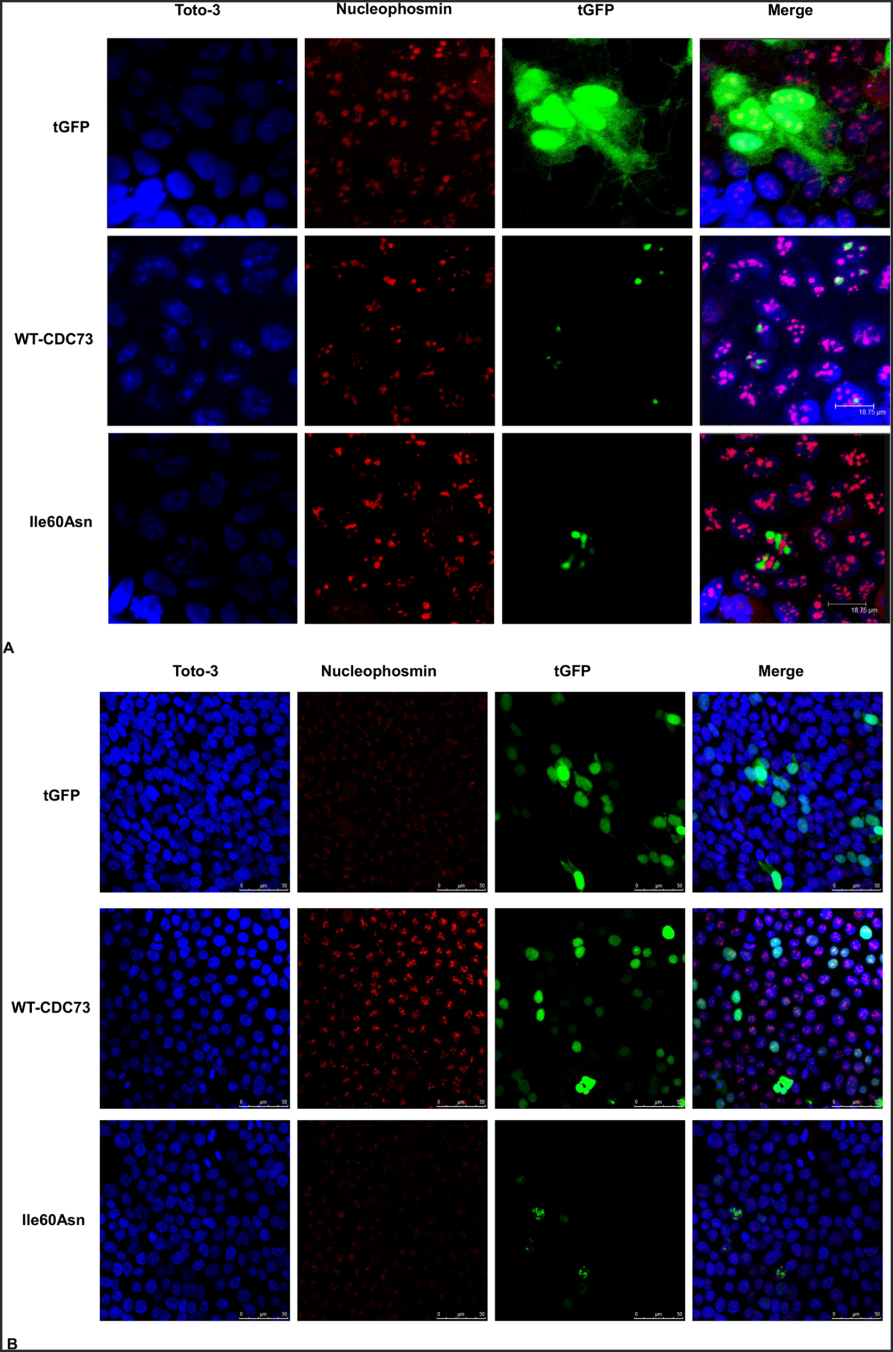
Co-localization of transfected parafibromin with nucleophosmin in HEK293A (A) and HeLa cells (B). Laser confocal microscopy showing the retention of nuclear expression and the lack of nucleolar accumulation of the parafibromin Ile60Asn mutant. HEK293A and HeLa cells were transfected with vectors encoding tGFP only (**tGFP**) or tGFP fusions with wild-type (**WT-CDC73**) or Ile60Asn parafibromin (**Ile60Asn**), and stained with anti-nucleophosmin antibody. An overlay (**Merge**) of the tGFP fluorescent signal with the nucleolar marker and the nuclear stain (**Toto-3**) is also shown.

To investigate whether the Ile60Asn mutation altered parafibromin cellular localization, vectors carrying wild-type and Ile60Asn mutant parafibromin, expressed as fusion proteins with tGFP at the C-terminus, were used in transfection experiments. Laser confocal imaging showed that HEK293A and HeLa cells transfected with wild-type-parafibromin exhibited a nuclear tGFP signal, with an evident protein concentration in the nucleolus, as demonstrated by co-localization with nucleophosmin (Figures 5A and 5B). By converse, cells transfected with Ile60Asn parafibromin showed a decrease in the nucleolar accumulation of the protein, even though the nuclear localization was retained (Figures 5A and 5B).

### The Ile60Asn Mutation Affects Cell Cycle Progression and Cell Proliferation

**Figure 6:**
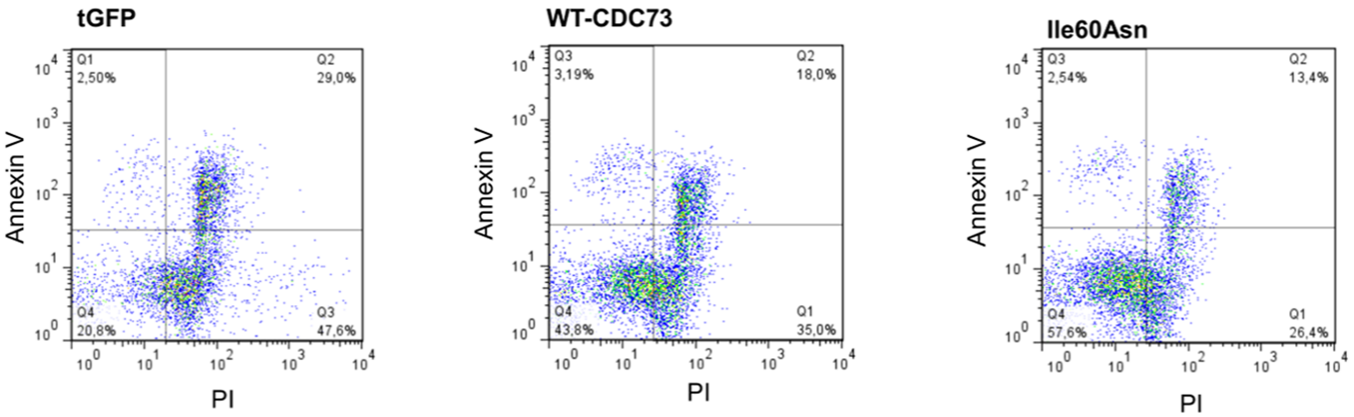
Flow cytometry analysis of apoptosis in HEK293A cells 48 hours after transfection. Representative experiment of apoptosis analysis performed through flow cytometric measurement of incorporation of propidium iodide (PI) and APC-conjugated- annexin V. Apoptosis has been evaluated in control cells (**tGFP**) and in cells transfected with both wild-type (**WT-CDC73**) and mutant parafibromin (**Ile60Asn**).

The flow cytometry analysis of APC-conjugated annexin V staining did not show any significant difference in the percentage of annexin-V positive cells neither between cells transfected with wild-type and mutant parafibromin (8.1±5.9% *vs.* 8.1±5.9%, *P* = 1), nor between control cells and Ile60Asn parafibromin-transfected cells (1.28±1.24% *vs.* 8.1±5.9%, *P* = 0.1). Wild-type parafibromin did not show a statistically significant difference in apoptosis induction with respect to control cells (8.1±5.9% *vs.* 1.28±1.24%, *P* = 0.1) (Figure 6).

**Figure 7:**
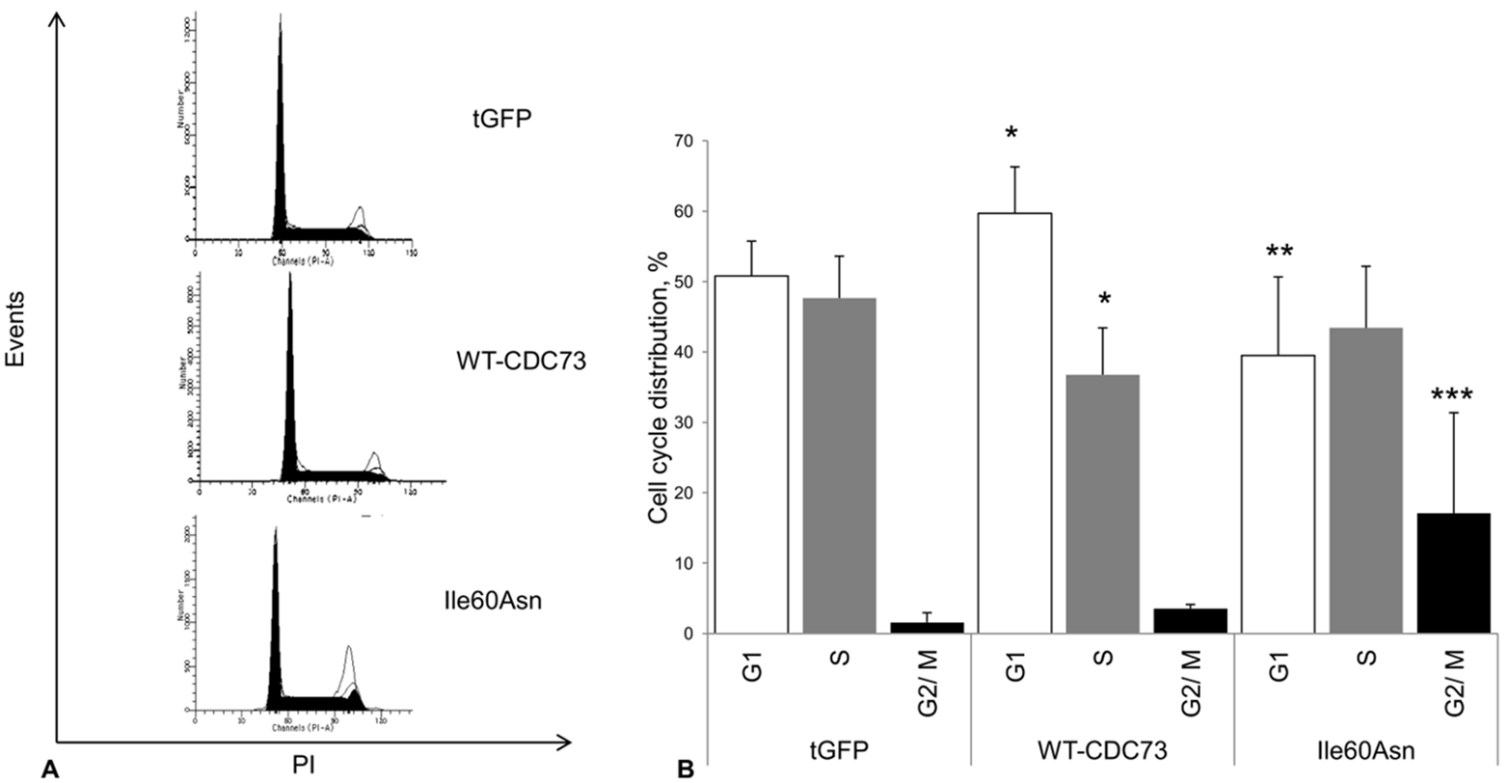
Flow cytometry analysis of cell cycle in transfected HEK293A cells. Cell cycle analysis with flow cytometry. HEK293A cells were transfected with the control vector (**tGFP**) or fusion proteins of tGFP with wild-type (**WT-CDC73**) or Ile60Asn mutant parafibromin (**Ile60Asn**), and the analysis was performed 48 hours after transfection. **A**. Flow cytometric analysis plotting cell number *vs.* intensity of propidium iodide (PI) signal from a representative experiment. **B**. Histograms showing the percentages of cells in the different phases of cell cycle. Values shown represent mean and standard deviations of pooled results from 4 independent experiments; *, *P*<0.05 compared to control cells; **, *P*<0.05 compared to wild-type parafibromin transfected cells; ***P<0.05 compared to both wild-type parafibromin-transfected cells and control cells.

To evaluate whether the Ile60Asn mutation impaired parafibromin antiproliferative activity, HEK293A cells were transfected with plasmid vectors encoding wild-type *CDC73* and the Ile60Asn mutant derivative as fused to tGFP, or tGFP only as a control and the effect of protein expression on cell cycle was analysed by cytofluorimetric analysis at 48 h post-transfection. Cells transfected with wild-type parafibromin exhibited an accumulation in G1 phase (59.7±6.6% *vs.* 50.8±4.9%, *P* = 0.027) and a decrease in the S phase (36.8±6.6% *vs.* 47.7±6%, *P* = 0.014) with respect to tGFP only expressing cells. Ile60Asn-transfected cells displayed a significant reduction in G1 with respect to wild-type parafibromin transfected cells (39.5±11.1% *vs.* 59.7±6.6%, *P* = 0.009) and an increase in G2/M phase as compared with cells transfected with the control vector (17.1±14.3% *vs.* 1.6±1.4%, *P* = 0.014) and wild-type parafibromin (17.1±14.3% *vs.* 3.5±0.6%, *P* = 0.009) (Figures 7A and 7B).

**Figure 8:**
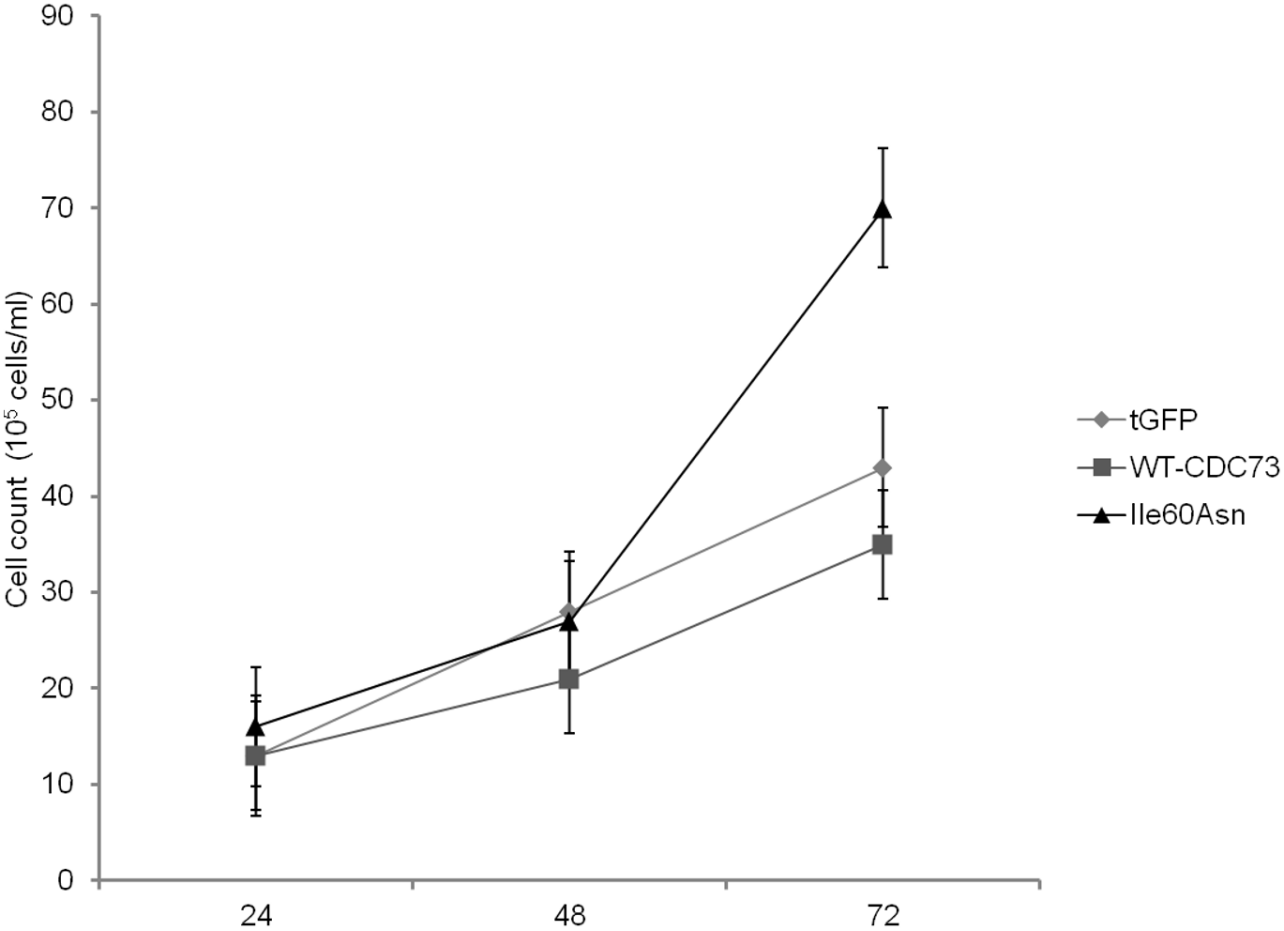
Cell proliferation assay. Cell counts at three different time points (24, 48 and 72 hours) after transfection of HeLa cells with Ile60Asn-tGFP-fused parafibromin (**Ile60Asn**), wild-type-tGFP-fused parafibromin (**WT-CDC73**) or tGFP-vector (**tGFP**).

In order to confirm the increase in the fraction of proliferating cells transfected with the Ile60Asn mutant, an analysis of cell proliferation was performed at 24, 48, and 72 h after transfection of HeLa cells. Our data showed that expression of wild-type *CDC73* decreased cell proliferation at each time point considered, while expression of the Ile60Asn mutant did not, rather resulting in increased proliferation of transfected cells when compared to control cells (Figure 8).

**Figure 9:**
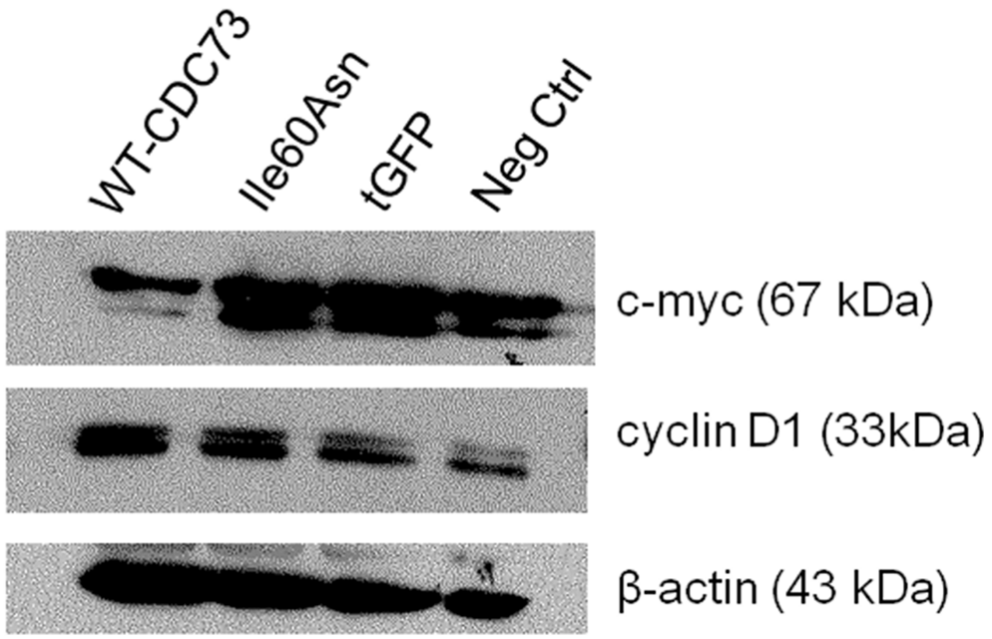
Western blotting for cyclin D1 and c-myc 48 hours post-transfection in HEK293A cells. Immunoblotting for cyclin D1 and c-myc in HEK293A cells transfected with Ile60Asn-tGFP-fused parafibromin (**Ile60Asn**), wild-type-tGFP-fused parafibromin (**WT-CDC73**) or tGFP-vector (**tGFP**). Nontransfected HEK293A cells (**Neg ctrl**) have been used as a negative control.

We also investigated if the Ile60Asn mutant could retain the capability to down-regulate the expression of two parafibromin transcriptional targets, i.e., cyclin D1 and c-myc, involved in the control of cell proliferation. Western blot analysis demonstrated that the levels of cyclin D1 (Figure 9) were comparable between control cells and cells overexpressing either wild-type or mutant parafibromin. By converse, while cells expressing wild-type protein exhibited a decrease in c-myc with respect to controls, cells expressing mutant parafibromin displayed c-myc levels comparable to control cells (Figure 9).

## Discussion

This study demonstrated, in an ossifying fibroma of the jaw arising in a HPT-JT patient, the presence of a somatic *CDC73* mutation that probably represented the “second hit” in tumor development, since it led to decreased parafibromin expression and loss of its nucleolar localization.

The mutation, *i.e.* Ile60Asn, which has not been described so far, affected a conserved amino acid in the N-terminal portion of parafibromin. The N-terminal domain is probably crucial for parafibromin activity and has been involved by most missense mutations of the *CDC73* gene [1], [11]. Among the reported missense mutations in the N-terminal domain of parafibromin, Leu64Pro impairs parafibromin anti-proliferative activity and ability to repress cyclin D1 expression [4]; Leu95Pro abolishes nucleolar localization and provides dominant interfering properties to parafibromin [17]; Arg77Pro causes cytoplasm retention, decreased half-life and cell overgrowth, even in the presence of endogenous parafibromin [18]. To the best of our knowledge, no other *CDC73* missense substitutions have been characterized so far.

The Ile60Asn mutation identified in this study had a high probability to be pathogenic, considering the high degree of conservation of the isoleucine at position 60. This mutation did not lead to a complete loss of parafibromin expression, as demonstrated by the retention of a faint but diffuse nuclear immunostaining in the ossifying fibroma. Experiments in cells transfected with wild-type and Ile60Asn mutant parafibromin demonstrated that the mutation determined a decreased stability of the protein, that may only partially be explained by an accelerated proteasomal degradation. Since proteasomal inhibition did not completely retrieve mutant parafibromin level, there are probably alternative mechanisms by which Ile60Asn parafibromin is degraded.

The subnuclear localization of Ile60Asn mutant and wild-type parafibromin was also evaluated, since there are evidences that parafibromin nucleolar accumulation has a key role for its tumor suppressor activity independently from nuclear distribution, as indicated by its loss in a subset of sporadic parathyroid carcinomas with *CDC73* mutations [19]. As demonstrated by confocal microscopy, the Ile60Asn mutant retained the nuclear expression, but showed a decrease in nucleolar accumulation.

The nucleolar distribution of parafibromin has been related to three nucleolar localization signals (NoLS) acting together, encompassing amino acids 76–92, 192–194, and 393–409 [20]. Although the Ile60Asn mutation is located 16 residues upstream the first NoLS, it caused loss of nucleolar localization.

Intriguingly, the Leu95Pro mutation, located outside the NoLS, has been reported to similarly impair nucleolar accumulation [17]. It is therefore conceivable that both Ile60Asn and Leu95Pro substitutions determine a change in the three-dimensional structure of parafibromin that impairs a combined action of the three NoLS.

The effect of parafibromin on cell apoptosis is controversial, since both pro-apoptotic [21] and anti-apoptotic [22] roles have been proposed. In our study, over-expression of both wild-type and mutant parafibromin had no effect on cell apoptosis, suggesting that the protein has probably no relevant pro- or anti-apoptotic activity in the cellular systems used.

The effect of wild-type and mutant parafibromin on cell cycle and cell proliferation was also assessed and, as expected for a tumor suppressor gene, an antiproliferative activity of wild-type parafibromin was demonstrated. Importantly, this activity was not retained by the Ile60Asn mutant, which stimulated cell proliferation, thus confirming previous findings about a dominant-interfering activity of other parafibromin mutants [17]–[18]. In addition, Ile60Asn mutant parafibromin lost the ability to down-regulate c-myc expression. These effects on cell proliferation and c-myc expression were probably the consequence of the low expression level and of the loss of nucleolar localization, that might be required to fully elicit parafibromin transcriptional functions. Since an intra-nuclear movement of yeast Cdc73 from the nucleoplasm toward the nucleolus has been observed in presence of mutant paf1 and rtf, both components of the Paf1 complex [23], we can speculate that the change in intra-nuclear parafibromin localization might in turn be due to a loss of interactions involving the N-terminal domain of parafibromin and unknown nucleolar partners.

In conclusion, the results of this study demonstrated that a novel missense mutation in the N-terminus of parafibromin stimulated cell proliferation and impaired parafibromin expression and nucleolar localization, contributing to the characterization of the N-terminal domain that is to date largely unknown and has probably a relevant role for parafibromin tumor suppressor function.
